# Nuclear volume effects in equilibrium stable isotope fractionations of mercury, thallium and lead

**DOI:** 10.1038/srep12626

**Published:** 2015-07-30

**Authors:** Sha Yang, Yun Liu

**Affiliations:** 1 State Key Laboratory of Ore Deposit Geochemistry, Institute of Geochemistry, Chinese Academy of Sciences, Guiyang 550002, China; 2 University of Chinese Academy of Sciences, Beijing 100049, China

## Abstract

The nuclear volume effects (NVEs) of Hg, Tl and Pb isotope systems are investigated with careful evaluation on quantum relativistic effects via the Dirac’s formalism of full-electron wave function. Equilibrium ^202^Hg/^198^Hg, ^205^Tl/^203^Tl, ^207^Pb/^206^Pb and ^208^Pb/^206^Pb isotope fractionations are found can be up to 3.61‰, 2.54‰, 1.48‰ and 3.72‰ at room temperature, respectively, larger than fractionations predicted by classical mass-dependent isotope fractionations theory. Moreover, the NVE can cause mass-independent fractionations (MIF) for odd-mass isotopes and even-mass isotopes. The plot of 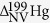
vs. 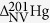 for Hg-bearing species falls into a straight line with the slope of 1.66, which is close to previous experimental results. For the first time, Pb^4+^-bearing species are found can enrich heavier Pb isotopes than Pb^2+^-bearing species to a surprising extent, e.g., the enrichment can be up to 4.34‰ in terms of ^208^Pb/^206^Pb at room temperature, due to their NVEs are in opposite directions. In contrast, fractionations among Pb^2+^-bearing species are trivial. Therefore, the large Pb fractionation changes provide a potential new tracer for redox conditions in young and closed geologic systems. The magnitudes of NVE-driven even-mass MIFs of Pb isotopes (i.e., 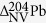) and odd-mass MIFs (i.e., 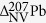) are almost the same but with opposite signs.

With rapid progresses in mass-spectrometer, great interests on stable isotope fractionations of heavy elements have been aroused. Evidences showed that heavy elements could have surprising isotopic fractionations as the consequence of the NVE^1^,^2^,^3^,^4^,^5^,^6^,^7^,^8^,^9^. The NVE is originated from differences in nuclear size and nuclear shape of isotopes^2^,^10^. It doesn’t belong to the well-known driving forces of equilibrium isotope fractionation, which are governed by the conventional Bigeleisen-Mayer theory^11^ or Urey method^12^.

The concept of NVE was proposed in spectrometric studies^10^. However, those early studies have not investigated its influences on isotopic fractionations. Fujii *et al*.^13^ found anomalous isotope fractionations in uranium isotope exchange experiments which violated the Bigeleisen-Mayer equation (or Urey model) but suggested the cause to be the difference in nuclear spin. Nishizawa *et al*.^1^ correctly interpreted the anomalous isotope effects of strontium by using isotope shift in atomic spectra (field shift). It was probably the first isotope NVE study. Hereafter, Bigeleisen^2^ and Nomura *et al*.^3^ independently recognized that those anomalous isotope fractionation phenomena, which were caused by the NVE, could lead to large stable isotope fractionations of heavy elements. Bigeleisen^2^ accordingly added the NVE as an important contribution into a modified calculation formula of the equilibrium isotope fractionation factor. He pointed out that the NVE is only a second order correction in chemical bonds, which suggested the NVE can have minor effect on vibrational frequencies^14^. Importantly, he emphasized that the NVE can change isotopic fractionation largely alone via the change of ground-state electronic energy.

Toshiyuki Fujii and his co-workers have made tremendous efforts on experimental evaluations of NVEs for isotope systems, including Ti, Sn, Zr, Ni, Zn, Gd, Nd, Cr, Sr, Te and Cd etc.^15^,^16^,^17^,^18^,^19^,^20^,^21^,^22^,^23^,^24^,^25^,^26^,^27^,^28^,^29^ Meanwhile, they performed quantum chemistry calculations for a few isotope systems, such as Zn^20^,^21^, Ni^18^, Tl^30^ and Pb^31^. Schauble^4^ used quantum chemistry methods to calculate NVEs of some heavy elements (e.g., Hg, Tl) and showed that the NVE could affect isotope fractionations of heavy elements to surprising degrees. Then, Abe *et al*.^5–7^ independently calculated the NVE-driven fractionation factors of U-bearing species. Schauble^32^ developed a new method to model nuclear volume effects in crystals. His new method was based on density functional theory (DFT), using the projector augmented wave method (DFT-PAW) with a three-dimensional periodic boundary condition for greater speed and compatibility.

In addition, Zheng *et al*.^33^ and Ghosh *et al*.^34^ did different experiments to estimate the NVE of mercury isotopes in the absence of light. They both assigned those mass-independent isotope fractionation signals as the consequence of the NVE. Wiederhold *et al*.^35^ also did experimental and theoretical investigations on Hg mass-indpendent isotope fractionations. Schauble^4^ and Wiederhold *et al*.^35^ have explored small NVE-driven Hg isotope fractionations in organic Hg-bearing species in depth. Moynier *et al*.^9^ reviewed the NVEs of Tl and U isotope systems in different natural environments, such as under low- or high-temperature conditions and in meteorites. The necessity of careful NVE evaluation during the exploration of new heavy elements is recognized by most people.

Right now, there are a few different computational methods used to investigate quantum relatistic effects associated with the NVE, e.g., Schauble^4^,^32^ used the DIRAC and ABINIT software package, Abe *et al*.^5^,^6^,^7^ used a four-component relativistic atomic program package-GRASP2K, Fujii *et al*.^18^,^20^,^21^,^30^,^31^ used a software provided by Tokyo University (UTchem). Recently, Nemoto *et al*.^36^ found a two-component realtivistic method (the finite-order Douglas-Kroll-Hess method with infinite-order spin-orbit interactions for the one-electron term and atomic-mean-field spin-same-orbit interaction for the two-electron term, i.e., IODKH-IOSO-MFSO) with almost equivalent accuracy but 30 times faster than the previous four-component method by DIRAC software package. They also predicted the IODKH-IOSO-MFSO method could compute larger system for future NVE calculation.

Here we calculate the NVE-driven fractionation factors of Hg-, Tl- and Pb-bearing species by using full-electron quantum chemistry calculation methods. Our method is similar to that of Schauble^4^, in which quantum relativistic effects have been carefully evaluated via four-component Dirac equation formalism^37^,^38^. Not only more new Hg- and Pb-bearing species (e.g., HgBr_4_^2^^−^, HgCl_3_^−^, HgBr_3_^−^ and many Pb^4+^-bearing species) are calculated here, but more mass-independent fractionations are investigated in light of recent findings on even-number Hg isotope MIFs^39^,^40^. Large fractionations (up to ca. 4‰ at room temperature) between Pb^4+^- and Pb^2+^-bearing species are found for the first time.

## Results

**Figure 1:**
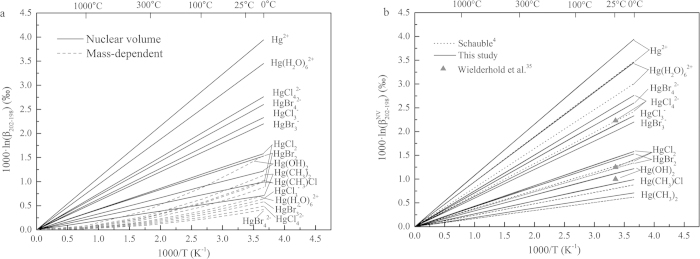
β-factors for Hg-bearing species relative to Hg^0^. (**a**) Contributions from CMDE and NVE, respectively. (**b**)Nuclear volume isotope fractionation factors (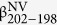-factors relative to Hg^0^ vapor) compared to the results of Schauble^4^ and Wielderhold *et al*.^35^.

**Figure 2:**
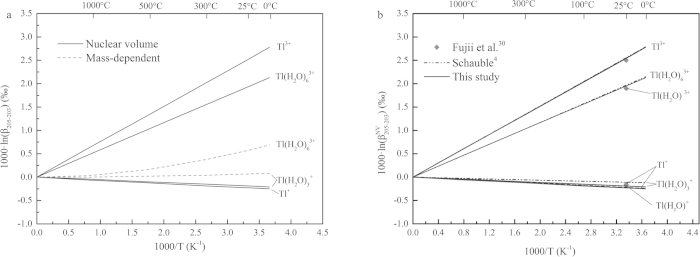
β-factors for Tl-bearing species relative to Tl^0^. (**a**) Contributions from CMDE and NVE, respectively. (**b**)Nuclear volume isotope fractionation factors (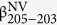-factors relative to Tl^0^) compared to the results of Schauble^4^ and Fujii *et al*.^30^.

**Figure 3:**
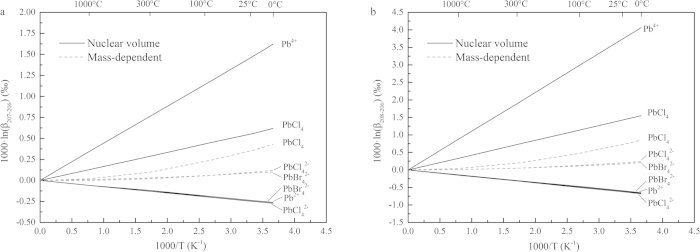
β-factors for Pb-bearing species relative to Pb^0^, including CMDE and NVE. (**a**) β_208-206_-factors of ^207^Pb/^206^Pb. (**b**) β_208-206_-factors of ^208^Pb/^206^Pb.

Equilibrium stable isotope fractionations of Hg-, Tl-, and Pb-bearing species are shown in Figures 1, 2, and 3 and Tables 1, 2, and 3 relative to Hg^0^, Tl^0^, and Pb^0^ in terms of 1000·lnβ, including conventional mass-dependent (1000·lnβ^MD^) and nuclear volume effect fractionation factors (1000·lnβ^NV^).

### Hg isotope system

NVEs alone can fractionate ^202^Hg/^198^Hg isotopes up to 3.61‰ at 25 °C. However, the largest classical mass-dependent fractionation are only 1.32‰ for ^202^Hg/^198^Hg at 25 °C. All Hg-bearing species enrich heavier isotope (^202^Hg) relative to Hg^0^ vapor. The NVE-driven isotope fractionations of inorganic species, such as Hg^2+^, HgCl_4_^2−^, HgBr_4_^2−^, HgCl_3_^−^, HgBr_3_^−^, HgCl_2_, HgBr_2_, Hg(H_2_O)_6_^2+^ or Hg(OH)_2_, are larger than those of organic molecules (e.g., Hg(CH_3_)Cl and Hg(CH_3_)_2_). On the contrary, CMDE fractionations of inorganic species are smaller than organic compounds except for Hg(OH)_2_.

### Tl isotope system

The NVE-driven fractionation of ^205^Tl/^203^Tl isotopes is up to 2.54‰ relative to Tl^0^ and the CMDE is only 0.58‰ for Tl(H_2_O)_6_^3+^ and 0.07‰ for Tl(H_2_O)_3_^+^ at 25 °C. Our NVE results show that Tl^3+^ ion and Tl^3+^-bearing compounds enriches heavier isotope (^205^Tl) relative to Tl^0^. However, Tl^+^ ion and Tl^+^-bearing compounds enriches lighter isotope (^203^Tl) compared to Tl^0^. Note that β-values of Tl^+^-bearing species are even smaller than the unity. This is because NVE tends to let heavier isotopes to be enriched in those atoms or ions with fewer s electrons or with more p, d and f electrons. Tl^0^ has more p electrons than Tl^+^-bearing species does.

### Pb isotope system

NVEs induce Pb isotope fractionations up to 1.48‰ (^207^Pb/^206^Pb) and 3.72‰ (^208^Pb/^206^Pb) relative to Pb^0^, at 25 °C. However, contributions from classical mass-dependent fractionation are small, about 0.1–0.4‰ for ^207^Pb/^206^Pb and 0–2–0.7‰ for ^208^Pb/^206^Pb at 25 °C. The isotope fractionations of Pb^4+^-bearing species (e.g., PbCl_4_) are larger than those of Pb^2+^-bearing species (e.g., PbCl_4_^2−^ or PbBr_4_^2−^) in terms of NVE or CMDE.

### Mass-independent fractionation of Hg and Pb isotopes

Table 4 shows NVE-driven mass-independent fractionations for ^199^Hg, ^200^Hg, and ^201^Hg isotopes at room temperature. Those MIFs are relative to the MIF of Hg vapor (i.e., Hg^0^). If the real MIF value of a specific Hg-bearing species is needed, one needs convert the number listed in Table 4 via the aid of experimental MIF data of Hg vapor. For example, according to Ghosh *et al*.^34^, the NVE-driven 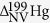 of Hg^0^ is about 0.14‰, therefore, 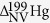 of Hg^2+^ should be −0.59‰ (i.e., −0.73‰ of Hg^2+^ listed in Table 4 plus 0.14‰).

**Figure 4:**
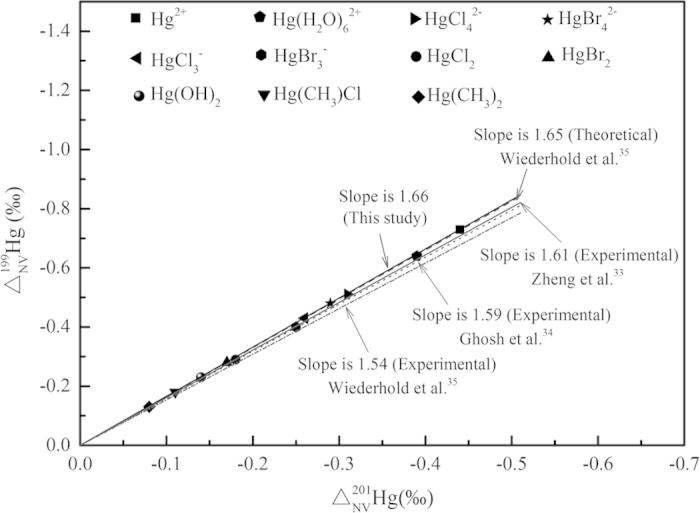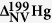 versus 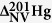 from NVE (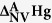-factors relative to Hg^0^ vapor) for Hg-bearing species at 25 °C. The black line is the slope of Δ^199^Hg/Δ^201^Hg of this study. The gray dash, dark gray solid, dark gray dot, dark gray short dash dot lines are the slop of Δ^199^Hg/Δ^201^Hg based on theoretical NVE-driven MIF calculated with radii of Landolt-Boernstein Databas^41^ (calculated by Wiederhold *et al*.^35^), experimental NVE-driven MIF of Zheng *et al*.^33^, Ghosh *et al*.^34^, Wiederhold *et al*.^35^, respectively.

All 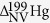 and 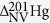 values listed in Table 4 except for Hg^0^ are negative. For all studied Hg species, the MIF ratio of two odd-mass isotopes (i.e., 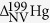/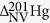) will fall on a straight line with the slope of 1.66 (Fig. 4), suggesting they will be changed in a proportional way. This result is almost identical to a previous theoretical result^35^ (i.e., with the slope of 1.65). This special relationship can be used to study MIFs caused by other reason via distinguishing the NVE signals from them.

Moreover, NVE can also cause mass-independent fractionations for odd-mass isotope (^207^Pb) and even-mass isotope (^204^Pb) (Table 5). The largest signals of NVE-driven MIF are up to −0.39‰ (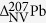) and 0.41‰ (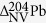) among all the studied species relative to Pb^0^ at 25 °C. The signs of even-mass isotope MIF (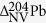) and odd-mass isotope MIF (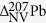) are opposite to each other although their magnitudes are almost the same (Table 5).

The calculation details, including optimized geometries, energies, harmonic vibrational frequencies, *et al*., have been documented in the Supplementary file for interested reader.

## Discussion

One of the special features of NVE is that it can cause large isotope fractionations between isolated atoms and ions (e.g., Hg^2+^-Hg^0^, Tl^3+^-Tl^+^ and Pb^4+^-Pb^2+^), which there would be no fractionation at all if based on the classical mass-dependent isotope fractionation theory, because there is no difference in terms of kinetic energies for them. Moreover, it seems that ions with more extra charges (e.g., with fewer s orbital electrons) can have larger NVEs and isotope fractionation potential than those with lesser charges (e.g., Tl^3+^ vs. Tl^+^, Pb^4+^ vs. Pb^2+^).

Comparing with previous studies (Fig. 1b and Table 1), our NVE-driven Hg isotope fractionation results are noticeably different from those of Schauble^4^ and Wiederhold *et al*.^35^. The NVE is proportional to difference in mean square nuclear charge radius of different nuclei (i.e., NVE ∝ δ<r^2^> and δ<r^2^> = <r^2^>_A_−<r^2^>_A′_), as King^10^ has pointed out based on spectrometric results. Therefore, we can explain the difference between Schauble^4^ and our results very clearly. Schauble^4^ used the nuclear charge radii of Angeli^42^ (i.e., <r^2^>^1/2^ of ^202^Hg and ^198^Hg are 5.4633fm and 5.4466fm) and the nuclear charge radius difference (δ<r^2^> = <r^2^>_A_−<r^2^>_A′_) is 0.182fm^2^. But we use the nuclear charge radii from Fricke and Heilig^41^ (i.e., <r^2^>^1/2^ of ^202^Hg and ^198^Hg are 5.462fm and 5.443fm) and the nuclear charge radius difference (δ<r^2^> = <r^2^>_A_−<r^2^>_A′_) is 0.207fm^2^. Our results are roughly 1.137 times of those of Schauble^4^, consistent with the radii difference ratio, i.e., 0.207/0.182 = 1.137.

We use the same mean square nuclare radii as Wiederhold *et al*.^35^, but different methods (i.e., DHF vs. MP2, respectively), which lead to different results. In addition, there are suggestions that calculated results of Hg-bearing species used the mean square nuclear charge radii of Fricke and Heilig^41^ are closer to the experiment results^35^. Note that different versions of the calculation software package (i.e., DIRAC04 and DIRAC13.1) have little impact on the calculated results (see Table S6).

Our NVE-driven Tl isotope fractionation results are in comparison with those of Schauble^4^ and Fujii *et al*.^30^ in terms of 1000·lnβ^NV^ (Fig. 2b and Table 2). Our results agree with those of Schauble^4^ perfectly because of using the similar methods and the same mean square nuclear charge radii (i.e., those radii from Angeli^42^). The only one exception is for the Tl(H_2_O)_3_^+^ case. The fractionations between Tl(H_2_O)_3_^+^ and Tl^0^ are larger than those of Schabule^4^, i.e., our result is −0.19‰ and their result is −0.11‰, at 25 °C. Our results are indeed very close to those of Fujii *et al*.^30^ with small differences (ca. 0.04–0.07‰).

Previous researches have shown mercury can undergo mass-dependent fractionation (MDF) as well as mass-independent fractionation (MIF) for odd-mass isotopes (Δ^199^Hg or Δ^201^Hg)^43^,^44^,^45^,^46^ and even-mass isotope (Δ^200^Hg)^39^,^40^. The mechanism leading to the even-number Hg isotope mass-independent fractionation is still unclear.

Gratz *et al*.^39^ firstly reported Δ^200^Hg in Great Lakes precipitation and ambient air up to 0.25‰. Later, Chen *et al*.^40^ found larger Δ^200^Hg in precipitation from Peterborough where is located in subarctic zone. They showed that snow samples obtained in winter have surprisingly large Δ^200^Hg values (up to 1.24‰) and rain water obtained in other seasons has much smaller Δ^200^Hg values (about 0.21‰ ~ 0.42‰). However, there is no convincing evidence can explain the even-mass number Hg MIF enigma.

With the calculation data from this study, we find that the NVE-driven 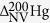 cannot be the reason to explain those even-mass number Hg MIF results. First, the magnitudes of NVE-driven 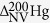 are much smaller than those found by Gratz *et al*.^39^ and Chen *et al*.^40^. Second, the sign of NVE-driven even-mass number Hg MIFs calculated here is opposite to those reported Δ^200^Hg results, meaning the NVE causes depletion of ^200^Hg instead of enrichment of ^200^Hg relative to ^198^Hg. Therefore, the observed large positive Δ^200^Hg signals must have other reasons or processes to be produced.

Because the half-life times of uranium isotopes are all very long, e.g., 4.5Ga for ^238^U and 0.7Ga for ^235^U, people actually treat uranium isotope system as a stable one in many young geologic systems^47^,^48^. As the decayed products of uranium, Pb isotope system can also be treated as a regular stable isotope system for young and closed geologic systems with homogenized formation processes. For example, in some rocks formed less than 10 million years (or younger), or in some plants, or in any system which is young and homogenized before its formation. The equilibrium Pb isotope fractionations between two compounds in such systems can be meaningful and useful. In such systems, the radiogenic Pb isotope differences are no longer existing but homogenized to a background value. For instance, a system with inherited very high ^208/206^Pb value has been homogenized in some processes. The compounds in such system will all have very high ^208/206^Pb values. Meanwhile, the small differences of ^208/206^Pb values among different compounds are caused by mass-driven and NVE-driven isotope fractionations. Our results can be used to explain such differences.

Fujii *et al*.^31^ firstly reported calculated NVE-driven Pb isotope fractionation factors for Pb^0^ and Pb^2+^-bearing species. We provide results of several new Pb-bearing species especially for Pb^4+^-bearing species. If comparing the NVE results between Pb^0^ and Pb^2+^ of Fujii *et al*.^31^ and ours, our results are marginally larger than theirs (^208^Pb/^206^Pb: 0.60‰ vs. 0.393‰ and ^207^Pb/^206^Pb: 0.24‰ vs. 0.156‰) due to different methods and software packages used. In general, Pb isotope fractionations among Pb^2+^-species are very small even with the driving force of NVE. However, we find surprisingly large fractionations (ca. 2 to 4‰) between Pb^4+^-bearing species and Pb^2+^-bearing species at room temperature. The fractionation magnitudes are similar or even larger than those Fe isotope fractionations between ferric and ferrous Fe-bearing species (e.g., Fe^3+^_(aq)_ vs. Fe^2+^_(aq)_) at low temperature, which have been broadly used as tracer for the change of redox conditions. Therefore, Pb isotope fractionations probably can also be used as a new tracer to study redox condition changes in young and closed geologic systems.

The occurrence of such large isotope fractionations is because the β-values of Pb^4+^-bearing and Pb^2+^-bearing species are in different directions, as the consequence of unique nuclear volume effects. Pb^4+^-bearing species enrich heavy isotopes relative to Pb^0^. However, Pb^2+^-bearing species enrich light isotope compared to Pb^0^, meaning β-values of Pb^2+^-bearing species are smaller than the unity. It is similar to the case of Tl^+^-bearing species. Pb^0^ is the one has more p electrons than Pb^2+^-bearing species. This finding cannot be explained if only based on classical isotope fractionation theory, which suggests all β values of any kind of isotope systems must be equal or larger than the unity.

## Conclusions

In this study, quantum chemical calculations (Dirac-Hartree-Fock) confirm that the nuclear volume effect plays a dominant role in equilibrium isotope fractionation for mercury, thallium and lead systems compared to the contributions of conventional mass-dependent effect, and agree with those conclusions of previous studies^4^,^30^,^31^. NVE-driven ^202^Hg/^198^Hg, ^205^Tl/^203^Tl, ^207^Pb/^206^Pb and ^208^Pb/^206^Pb fractionations for Hg-, Tl- and Pb-bearing species can be up to 3.61‰, 2.54‰, 1.48‰ and 3.72‰ at 25 °C, respectively. Moreover, the NVE-driven mass-independent fractionations of ^199^Hg are larger than those of ^201^Hg and ^200^Hg which is up to −0.73‰. The ratio of 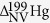/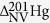
is 1.66 which agrees well with previous experimental and theoretical results. Furthermore, those NVE-driven MIFs of ^200^Hg calculated here are not only too small to be compared with the Δ^200^Hg results reported in snow and water samples^39^,^40^, but also with the opposite sign, meaning the NVE is not the reason of those Δ^200^Hg signals.

Surprisingly, we find Pb isotope fractionations between Pb^4+^-bearing and Pb^2+^-bearing species can be up to 2 - 4‰ at room temperatures, suggesting a potential new tracer for redox condition changes in young and closed geologic systems. The NVE-driven MIFs of 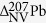 and 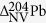 are with moderate magnitudes but in opposite signs (i.e., 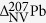 ≈ −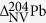).

## Methods

### Conventional mass-dependent effect (CMDE)

Bigeleisen and Mayer^11^ and Urey^12^ suggested a well-known method for calculating the isotope fractionation factor, which is called the Bigeleisen-Mayer equation (hereafter B-M equation) or the Urey model. The B-M equation was based on the Born-Oppenheimer and harmonic approximations. According to the B-M equation, the natural logarithm of the isotope fractionation factor for an isotope exchange reaction under high-temperature approximations is


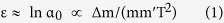


where ε is the isotope enrichment factor and is roughly equal to lnα_0_; α_0_ is the isotope fractionation factor; m and m′ are the masses of the heavy and light isotopes, respectively; Δm is the relative mass difference of isotopes (i.e., Δm=m-m′). When the temperature is constant, enrichment factor is proportional to Δm/mm′. According to this equation, the isotope fractionation of heavy elements (e.g., Hg, Tl or Pb) would be small.

For an exchange reaction A′Y + AX = A′X + AY, the equilibrium CMDE fractionation factors is calculated^11^,^12^


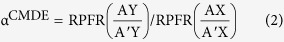


where RPFR is the reduced partition function ratio and it is expressed in term of the harmonic vibrational frequencies with isotope substitution


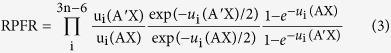


where A and A′ are the heavy and light isotopes of the element A; u_i_(AX) = h*v*_i_(AX)/kT; *v*_i_(AX) is the *i*th harmonic vibrational frequency of AX molecule; h and k are Planck and Boltzmann constant; T is the absolute temperature.

### Nuclear volume effect (NVE)

Based on spectrometric results, King^10^ proposed that the NVE was proportional to difference in mean square nuclear charge radius of different nuclei (i.e., NVE ∝ δ<r^2^> and δ <r^2^> = <r^2^>_A_− <r^2^>_A′_). Upon the inspiration of U isotope exchange experiments, Bigeleisen^2^ revised the B-M equation and added the NVE term into it. The logarithm of the corrected isotope fractionation factor became


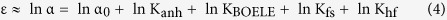


where lnα_0_ is the isotope fractionation factor under the B-M equation approximations; lnK_anh_ is the anharmonic correction term; lnK_BOELE_ is the correction to the Born-Oppenheimer approximation; lnK_fs_ is the NVE term (also called nuclear field shift); lnK_hf_ is the term for nuclear spin effect. In the terminology of Bigeleisen, the nuclear field shift actually includes both shape and size effects. However, the contribution from nuclear size is easy to calculate but that from nuclear shape is very difficult to evaluate and trivial. Therefore, people trend to use NVE instead of nuclear field shift for more precise description^4^.

Because of extremely small anharmonic corrections for heavy elements, lnK_anh_ can be safely neglected^2^. The correction to the Born-Oppenheimer approximation is related to Δm/mm′^49^,^50^. Therefore, lnα_0_ and lnK_BOELE_ are both proportional to Δm/mm′ when temperature is a constant. Based on the investigations on U isotope exchange reactions, Bigeleisen^2^ showed that nuclear spin effect was also very small and could be safely neglected.

Because the NVE is related to the difference in ground-state electronic energies, it can be written as^2^


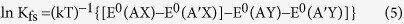


where E^0^ is the ground-state electronic energy; AX and A’X represent different isotopologues; k is the Boltzmann’s constant and T is in absolute temperature (K). We can see the magnitude of NVE is proportional to 1/T and to ground-state electronic energy differences due to isotopic substitutions.

### Mass-independent fractionation (MIF)

Here we use Hg isotopes as an example to introduce the concept of mass-independent isotope fractionation (MIF). If we define δ^A^Hg as


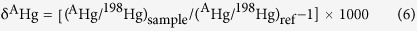


Then the mass-independent isotope fractionation (MIF) of any pair of Hg isotopes (e.g., ^A^Hg/^198^Hg) will be


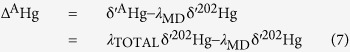


where 
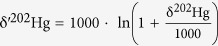
51,52, λ_TOTAL_ includes λ_MD_ (the conventional mass-dependent scaling factor), λ_NV_ (the nuclear volume scaling factor), λ_MIE_ (the magnetic isotope effect scaling factor) and other scaling factors. And if we just consider the MIF caused by the NVE, it would be


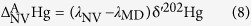


where λ_MD_ is actually calculated using the high temperature approximation of equilibrium fractionation^52^


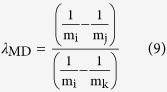


This is because λ_MD_ values for heavy metal isotope systems are only weakly temperature-dependent^53^.

λ_NV_ is calculated from the mean square nuclear charge radii^4^


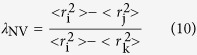


where m_i_, m_j_ and m_k_ are the masses of isotopes i, j and k, respectively; 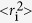, 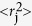
and 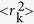 are their mean square nuclear charge radii.

Unfortunately, 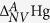 cannot be calculated theoretically because the value of δ^202^Hg for a specific Hg species is unknown. Instead, we calculate the relative MIF in comparison of Hg vapor (Hg^0^):


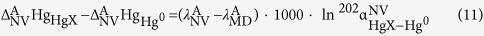


If experimental results of MIF for Hg^0^ vapor caused by NVE are available, we can obtain MIFs of other Hg species through “equation (11)”. For example, Ghosh *et al*.^34^ observed MIFs for odd isotopes (^199^Hg and ^201^Hg) and small MIFs for even isotope (^200^Hg) in the vapor phase (Hg^0^) caused by NVE at room temperature. Their average 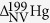, 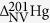 and 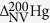 values for Hg^0^ were 0.14 ± 0.01‰, 0.09 ± 0.01‰ and 0.01 ± 0.03‰, respectively. The results of equilibrium evaporation experiments of Estrade *et al*.^45^ were similar to those of Ghosh *et al*.^34^ and their 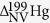, 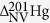 and 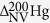 values for Hg^0^ were 0.12‰, 0.07‰ and 0.01‰, respectively, in the temperature range of 2–22 °C.

### Computational quantum chemistry methods

Ground-state electronic energies calculations are performed with DIRAC13.1 software package^54^. All-electron Dirac–Hartree–Fock (DHF) theory is used to calculate relativistic electronic structures of Hg-, Tl- and Pb-bearing species with four-component wave functions. Our calculation details are similar to those of Schauble^4^. “Double-zeta” basis sets^55^,^56^ are used for Hg, Tl and Pb atoms and uncontracted cc-pVDZ basis sets^57^ are used for other light atoms (H, O, C, Cl and Br). The molecular geometries were firstly optimized at pseudo-potential HF calculations (by Gaussian 03 software^58^) as initial guesses. Following the methods of Schauble^4^, we optimize structures by using the iteratively quadratic fitting method (i.e., energy vs. bond-length fitting) instead of free geometry optimization using Dirac 13.1 to save computing time. Hg(H_2_O)_6_^2+^ (T_h_), HgCl_4_^2−^ (T_d_), Tl(H_2_O)_6_^2+^ (T_h_), Tl(H_2_O)_3_^+^ (C_3_) and PbCl_4_^2−^ (T_d_) are chosen to compare their results calculated by the iteratively quadratic fitting method and by the free optimization method. The results show that these two methods can produce almost identical geometries but the former consumes much lesser time.

After geometry optimization, all Hg-, Tl- and Pb-bearing species are calculated for obtaining their ground-state electronic energies by using DIRAC 13.1. Different isotopologues will use their own Gaussian exponent ξ as in this form^59^:


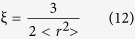


where the mean square nuclear charge radii (<r^2^>) can be found from the Landolt-Boernstein Database^41^ for Hg and from Angeli^42^ for Tl and Pb.

Different from closed shell species, we also use the complete open shell configuration interaction (COSCI) method to calculate the ground-state energies of opened shell species (Tl^0^ and Pb^0^ with the electron configuration as [Xe]4f^14^5d^10^6s^2^6p^1^ and [Xe]4f^14^5d^10^6s^2^6p^2^, respectively).

With the calculated ground-state electronic energies, the NVE can be calculated from “equation (5)”. For example, the NVE on isotope fractionation of an HgX-Hg^0^ isotope exchange reaction is^4^


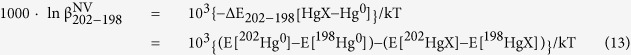


where the β(X) factor is the equilibrium fractionation factor between substance X and an ideal monoatomic gas^60^.

The molecular geometries and harmonic interatomic vibrational frequency are calculated at pseudo-potential Hartree-Fock (HF) level by Guassian03 software package^58^. We treat inner-shell electrons of Hg, Tl, and Pb atom by using relativistic pseudo-potentials. However, valance and intermediate-shell electrons are treated with a double-zeta basis sets (cc-pVDZ–PP) and cc-pVDZ basis sets are used for H, C, O, Cl and Br atoms.

The usual isotope fractionation between substance A and substance B is defined as


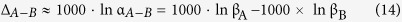


where α is the equilibrium isotope fractionation factor.

## Additional Information

**How to cite this article**: Yang, S. and Liu, Y. Nuclear volume effects in equilibrium stable isotope fractionations of mercury, thallium and lead. *Sci. Rep*. **5**, 12626; doi: 10.1038/srep12626 (2015).

## Supplementary Material

Supplementary Information

## Figures and Tables

**Table 1:** Calculated stable isotope fractionation factors for Hg-bearing species relative to Hg^0^ (in per mil), including conventional mass-dependent effect (CMDE) fractionation factors ( 
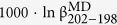
) and nuclear volume effect (NVE) fractionation factors (
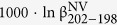
 ).

	This study^a^)					Schauble^b^)					Wielderhold *et al*.^c^)
	0 °C	25 °C	100 °C	300 °C	1000 °C	0 °C	25 °C	100 °C	300 °C	1000 °C	25 °C
CMDE fractionation factor ( 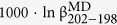 )											
Hg^0^	0	0	0	0	0	0	0	0	0	0	
Hg^2+^	0	0	0	0	0	0	0	0	0	0	
HgCl_2_	1.00	0.84	0.55	0.24	0.05	1.08	0.92 ± 0.1	0.60	0.26	0.05	0.84
HgBr_2_	0.88	0.74	0.48	0.21	0.04	0.95	0.80 ± 0.1	0.51	0.22	0.04	
Hg(CH_3_)Cl	1.02	0.87	0.57	0.25	0.05	1.04	0.89 ± 0.1	0.58	0.25	0.05	
Hg(CH_3_)_2_	1.13	0.97	0.64	0.28	0.06	1.14	0.97 ± 0.1	0.65	0.29	0.06	
HgCl_3_^−^	0.64	0.54	0.35	0.15	0.03						
HgCl_4_^2−^	0.49	0.41	0.26	0.11	0.02	0.67	0.56+0.7/−0.1	0.36	0.15	0.03	0.40
HgBr_3_^−^	0.58	0.49	0.31	0.13	0.03						
HgBr_4_^2−^	0.42	0.35	0.22	0.09	0.02						
Hg(H_2_O)_6_^2+^	0.71	0.60	0.39	0.17	0.03	1.13	0.96 ± 0.4	0.62	0.27	0.05	
Hg(OH)_2_	1.54	1.32	0.88	0.39	0.08						1.19
NVE fractionation factor ( 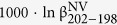 )											
Hg^0^	0	0	0	0	0	0	0	0	0	0	
Hg^2+^	3.94	3.61	2.89	1.88	0.85	3.47	3.17 ± 0.6	2.54	1.65	0.74	
HgCl_2_	1.58	1.45	1.16	0.75	0.34	1.39	1.27 ± 0.3	1.02	0.66	0.30	1.25
HgBr_2_	1.53	1.40	1.12	0.73	0.33	1.34	1.23 ± 0.2	0.98	0.64	0.29	
Hg(CH_3_)Cl	0.99	0.91	0.73	0.47	0.21	0.88	0.80 ± 0.2	0.64	0.42	0.19	
Hg(CH_3_)_2_	0.71	0.65	0.52	0.34	0.15	0.62	0.57 ± 0.1	0.45	0.30	0.13	
HgCl_3_^−^	2.33	2.14	1.71	1.11	0.50						
HgCl_4_^2−^	2.76	2.53	2.02	1.31	0.59	2.42	2.22 ± 0.4	1.77	1.16	0.52	2.23
HgBr_3_^−^	2.20	2.01	1.61	1.05	0.47						
HgBr_4_^2−^	2.60	2.38	1.91	1.24	0.56						
Hg(H_2_O)_6_^2+^	3.45	3.16	2.52	1.64	0.74	3.01	2.75 ± 0.6	2.20	1.43	0.64	
Hg(OH)_2_	1.22	1.12	0.90	0.58	0.26						1.00
Total fractionation factor ( 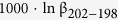 )											
Hg^0^	0	0	0	0	0	0	0	0	0	0	
Hg^2+^	3.94	3.61	2.89	1.88	0.85	3.47	3.17 ± 0.6	2.54	1.65	0.74	
HgCl_2_	2.58	2.29	1.71	0.99	0.39	2.47	2.19 ± 0.3	1.62	0.92	0.35	2.09
HgBr_2_	2.41	2.14	1.60	0.94	0.37	2.29	2.03 ± 0.2	1.49	0.86	0.33	
Hg(CH_3_)Cl	2.01	1.78	1.30	0.72	0.26	1.92	1.69 ± 0.2	1.22	0.67	0.24	
Hg(CH_3_)_2_	1.84	1.62	1.16	0.62	0.21	1.76	1.54 ± 0.1	1.10	0.59	0.19	
HgCl_3_^−^	2.97	2.68	2.06	1.26	0.53						
HgCl_4_^2−^	3.25	2.94	2.28	1.42	0.61	3.09	2.78+0.8/0.4	2.13	1.31	0.55	2.63
HgBr_3_^−^	2.78	2.50	1.92	1.18	0.50						
HgBr_4_^2−^	3.02	2.73	2.13	1.33	0.58						
Hg(H_2_O)_6_^2+^	4.16	3.76	2.91	1.81	0.77	4.14	3.71 ± 0.7	2.82	1.70	0.69	
Hg(OH)_2_	2.76	2.44	1.78	0.97	0.34						2.19

^a^Calculated with <r^2^> values of Fricke and Heilig^41^ by using the software package DIRAC13.1.

^b^Calculated with <r^2^> values of Angeli^42^ by using the software package DIRAC04 by Schauble^4^.

^c^Calculated with <r^2^> values of Fricke and Heilig^41^ by using the software package DIRAC08 by Wiederhold *et al*.^35^.

**Table 2:** Calculated stable isotope fractionation factors for Tl-bearing species relative to Tl^0^ (in per mil), including conventional mass-dependent effect (CMDE) fractionation factors (
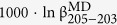
) and nuclear volume effect (NVE) fractionation factors (
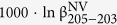
 ).

	This study					Schauble^a^)					Fujii *et al*.^b^)
	0 °C	25 °C	100 °C	300 °C	1000 °C	0 °C	25 °C	100 °C	300 °C	1000 °C	25 °C
CMDE fractionation factor ( 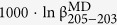 )											
Tl^0^	0	0	0	0	0	0	0	0	0	0	
Tl^+^	0	0	0	0	0	0	0	0	0	0	
Tl^3+^	0	0	0	0	0	0	0	0	0	0	
Tl(H_2_O)_3_^+^	0.08	0.07	0.04	0.02	0.00	0.08	0.07 ± 0.1	0.04	0.02	0.00	0.063^c)^
Tl(H_2_O)_6_^3+^	0.69	0.58	0.38	0.16	0.03	0.77	0.65 ± 0.2	0.44	0.18	0.04	0.423
NVE fractionation factor ( 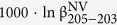 )											
Tl^0^	0	0	0	0	0	0	0	0	0	0	
Tl^+^	−0.25	−0.23	−0.19	−0.12	−0.06	−0.24	−0.22 ± 0.04	−0.18	−0.11	−0.05	−0.157
Tl^3+^	2.78	2.54	2.03	1.32	0.60	2.79	2.55 ± 0.5	2.04	1.32	0.60	2.501
Tl(H_2_O)_3_^+^	−0.21	−0.19	−0.15	−0.10	−0.04	−0.12	−0.11 ± 0.02	−0.09	−0.06	−0.03	−0.168^d^)
Tl(H_2_O)_6_^3+^	2.13	1.95	1.56	1.02	0.46	2.15	1.97 ± 0.4	1.57	1.02	0.46	1.898^e^)
Total fractionation factor ( 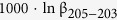 )											
Tl^0^	0	0	0	0	0	0	0	0	0	0	
Tl^+^	−0.25	−0.23	−0.19	−0.12	−0.06	−0.24	−0.22 ± 0.04	−0.18	−0.11	−0.05	
Tl^3+^	2.78	2.54	2.03	1.32	0.60	2.79	2.55 ± 0.5	2.04	1.32	0.60	
Tl(H_2_O)_3_^+^	−0.13	−0.12	−0.11	−0.08	−0.04	−0.04	−0.04 ± 0.1	−0.05	−0.04	−0.03	
Tl(H_2_O)_6_^3+^	2.82	2.53	1.94	1.18	0.49	2.92	2.62 ± 0.4	2.01	1.20	0.50	

^a^Calculated by Schauble^4^ using the software package DIRAC04.

^b^Calculated by Fujii *et al*.^30^ using UTChem program at 298 K.

^c^Calculated for Tl(H_2_O)_6_^+^.

^d^Calculated for Tl(H_2_O)^+^.

^e^Calculated for Tl(H_2_O)^3+^.

**Table 3:** Calculated stable isotope fractionation factors for Pb-bearing species relative to Pb^0^ (in per mil), including conventional mass-dependent effect (CMDE) and nuclear volume effect (NVE).

species	0 °C	25 °C	100 °C	300 °C	1000 °C	0 °C	25 °C	100 °C	300 °C	1000 °C
	CMDE fractionation factor ( 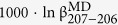 )					CMDE fractionation factor ( 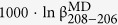 )				
Pb^0^	0	0	0	0	0	0	0	0	0	0
Pb^2+^	0	0	0	0	0	0	0	0	0	0
Pb^4+^	0	0	0	0	0	0	0	0	0	0
PbCl_4_^2−^	0.12	0.10	0.06	0.03	0.01	0.24	0.20	0.13	0.05	0.01
PbBr_4_^2−^	0.10	0.09	0.06	0.02	0.00	0.21	0.17	0.11	0.05	0.01
PbCl_4_	0.43	0.36	0.24	0.10	0.02	0.85	0.72	0.47	0.20	0.04
	NVE fractionation factor ( 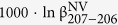 )					NVE fractionation factor ( 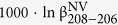 )				
Pb^0^	0	0	0	0	0	0	0	0	0	0
Pb^2+^	−0.26	−0.24	−0.19	−0.13	−0.06	−0.66	−0.60	−0.48	−0.31	−0.14
Pb^4+^	1.61	1.48	1.18	0.77	0.35	4.06	3.72	2.97	1.93	0.87
PbCl_4_^2−^	−0.27	−0.25	−0.20	−0.13	−0.06	−0.68	−0.62	−0.50	−0.32	−0.15
PbBr_4_^2−^	−0.26	−0.24	−0.19	−0.12	−0.06	−0.65	−0.59	−0.47	−0.31	−0.14
PbCl_4_	0.62	0.56	0.45	0.29	0.13	1.55	1.42	1.13	0.74	0.33
	Total fractionation factor ( 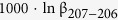 )					Total fractionation factor ( 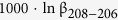 )				
Pb^0^	0	0	0	0	0	0	0	0	0	0
Pb^2+^	−0.26	−0.24	−0.19	−0.13	−0.06	−0.66	−0.60	−0.48	−0.31	−0.14
Pb^4+^	1.61	1.48	1.18	0.77	0.35	4.06	3.72	2.97	1.93	0.87
PbCl_4_^2−^	−0.15	−0.15	−0.14	−0.10	−0.05	−0.44	−0.42	−0.37	−0.27	−0.14
PbBr_4_^2−^	−0.16	−0.15	−0.13	−0.10	−0.06	−0.44	−0.42	−0.36	−0.26	−0.13
PbCl_4_	1.05	0.92	0.69	0.39	0.15	2.40	2.14	1.60	0.94	0.37

**Table 4:** Conventional mass-dependent (CMD) and nuclear volume (NV) scaling factors and 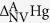 values for different Hg-bearing species relative to Hg^0^ at 25 °C.

	^198^Hg/^198^Hg	^199^Hg/^198^Hg	^200^Hg/^198^Hg	^201^Hg/^198^Hg	^202^Hg/^198^Hg
NV scaling factor	0.0	0.0525	0.4732	0.6312	1.0
CMD scaling factor	0.0	0.2539	0.5049	0.7539	1.0
	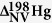	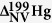	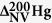	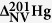	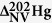
Hg^0^	0.0	0.0	0.0	0.0	0.0
Hg^2+^	0.0	−0.73	−0.11	−0.44	0.0
HgCl_2_	0.0	−0.29	−0.05	−0.18	0.0
HgBr_2_	0.0	−0.28	−0.04	−0.17	0.0
Hg(CH_3_)Cl	0.0	−0.18	−0.03	−0.11	0.0
Hg(CH_3_)_2_	0.0	−0.13	−0.02	−0.08	0.0
HgCl_3_^−^	0.0	−0.43	−0.07	−0.26	0.0
HgCl_4_^2−^	0.0	−0.51	−0.08	−0.31	0.0
HgBr_3_^−^	0.0	−0.40	−0.06	−0.25	0.0
HgBr_4_^2−^	0.0	−0.48	−0.08	−0.29	0.0
Hg(H_2_O)_6_^2+^	0.0	−0.64	−0.10	−0.39	0.0
Hg(OH)_2_	0.0	−0.23	−0.04	−0.14	0.0

**Table 5:** Conventional mass-dependent (CMD) and nuclear volume (NV) scaling factors and 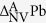 values for different Pb-bearing species relative to Pb^0^ at 25 °C.

	^204^Pb/^206^Pb	^206^Pb/^206^Pb	^207^Pb/^206^Pb	^208^Pb/^206^Pb
NV scaling factor	−0.9097	0.0	0.3980	1.0
CMD scaling factor	−1.0193	0.0	0.5026	1.0
	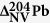	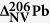	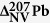	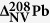
Pb^0^	0.0	0.0	0.0	0.0
Pb^2+^	−0.07	0.0	0.06	0.0
Pb^4+^	0.41	0.0	−0.39	0.0
PbCl_4_^2−^	−0.07	0.0	0.06	0.0
PbBr_4_^2−^	−0.06	0.0	0.06	0.0
PbCl_4_	0.16	0.0	−0.15	0.0

## References

[b1] Nishizawa K., Satoyama T., Miki T. & Yamamoto T. Strontium isotope effect in liquid-liquid extraction of strontium chloride using a crown ether. J. Nucl. Sci. Technol. 32, 1230–1235 (1995).

[b2] Bigeleisen J. Nuclear size and shape effects in chemical reactions. Isotope chemistry of the heavy elements. J. Am. Chem. Soc. 118, 3676–3680 (1996).

[b3] Nomura M., Higuchi N. & Fujii Y. Mass dependence of uranium isotope effects in the U(IV)−U(VI) exchange reaction. J. Am. Chem. Soc. 118, 9127–9130 (1996).

[b4] Schauble E. A. Role of nuclear volume in driving equilibrium stable isotope fractionation of mercury, thallium, and other very heavy elements. Geochim. Cosmochim. Acta 71, 2170–2189 (2007).

[b5] Abe M., Suzuki T., Fuji Y. & Hada M. An ab initio study based on a finite nucleus model for isotope fractionation in the U(III)-U(IV) exchange reaction system. J. Chem. Phys. 128, 144309-1–144309-6 (2008). 18412447 10.1063/1.2898541

[b6] Abe M., Suzuki T., Fujii Y., Hada M. & Hirao K. An ab initio molecular orbital study of the nuclear volume effects in uranium isotope fractionations. J. Chem. Phys. 129, 164309-1–164309-7 (2008). 19045268 10.1063/1.2992616

[b7] Abe M., Suzuki T., Fujii Y., Hada M. & Hirao K. Ligand effect on uranium isotope fractionations caused by nuclear volume effects: An ab initio relativistic molecular orbital study. J. Chem. Phys. 133, 044309-1–044309-5 (2010). 20687652 10.1063/1.3463797

[b8] Fujii T., Moynier F. & Albarede F. The nuclear field shift effect in chemical exchange reactions. Chem. Geol. 267, 139–156 (2009).

[b9] Moynier F., Fujii T., Brennecka G.A. & Nielsen S.G. Nuclear field shift in natural environments. C. R. Geosci. 345, 150–159 (2013).

[b10] King W. H. Isotope shifts in atomic spectra 1st edn (eds Burke P. G. .) Ch. 4, 35–53 (New York: Plenum Press, 1984).

[b11] Bigeleisen J. & Mayer M. G. Calculation of equilibrium constants for isotopic exchange reactions. J. Chem. Phys. 15, 261–267 (1947).

[b12] Urey H. C. The thermodynamic properties of isotopic substances. J. Chem. Phys. (London) 562–581 (1947). 10.1039/jr9470000562 20249764

[b13] Fujii Y., Nomura M., Onitsuka H. & Takeda K. Anomalous isotope fractionation in uranium enrichment process. J. Nucl. Sci. Technol. 26, 1061–1064 (1989).

[b14] Bigeleisen J. Second-order correction to the Bigeleisen–Mayer equation due to the nuclear field shift. Proc. Natl. Acad. Sci. USA 95, 4808–4809 (1998). 9560183 10.1073/pnas.95.9.4808 PMC20168

[b15] Fujii T., Inagawa J. & Nishizawa K. Influences of nuclear mass, size, shape and spin on chemical isotope effect of titanium. Ber Burtsenges. Phys. Chem. 102, 1880–1885 (1998).

[b16] Moynier F., Fujii T. & Telouk P. Mass-independent isotopic fractionation of tin in chemical exchange reaction using a crown ether. Anal. Chim. Acta 632, 234–239 (2009). 19110099 10.1016/j.aca.2008.11.015

[b17] Fujii T., Yamamoto T., Inagawa J., Watanabe K. & Nishizawa K. Influences of nuclear size and shape and nuclear spin on chemical isotope effect of zirconium-crown complex. Ber Burtsenges. Phys. Chem. 102, 663–669 (1998).

[b18] Fujii T., Moynier F., Dauphas N. & Abe M. Theoretical and experimental investigation of nickel isotopic fractionation in species relevant to modern and ancient oceans. Geochim. Cosmochim. Acta 75, 469–482 (2011).

[b19] Nishizawa K. . Contributions of nuclear size and shape, nuclear mass, and nuclear spin to enrichment factors of zinc isotopes in a chemical exchange reaction by a cryptand. Sep. Sci. Technol. 33, 2101–2112 (1998).

[b20] Fujii T. . Mass-dependent and Mass-independent isotope effects of zinc in a redox reaction. J. Chem. Phys. A 113, 12225–12232 (2009). 10.1021/jp904882d 19807141

[b21] Fujii T., Moynier F., Telouk P. & Abe M. Experimental and theoretical investigation of isotope fractionation of zinc between aqua, chloro, and macrocyclic complexes. J. Phys. Chem. A 114, 2543–2552 (2010). 20121110 10.1021/jp908642f

[b22] Fujii T. . Nuclear size and shape effect in chemical isotope effect of gadolinium using dicyclohexano-18-crown-6. Solvent Extr. Ion Exch. 17, 1219–1229 (1999).

[b23] Fujii T. . Nuclear size and shape effects in chemical isotope enrichment of neodymium using a crown ether. Solvent Extr. Ion Exch. 18, 1155–1166 (2000).

[b24] Fujii T. . Nuclear field shift effect in the isotope exchange reaction of chromium(III) using a crown ether. J. Phys. Chem. A 106, 6911–6914 (2002).

[b25] Fujii T., Suzuki D. & Yamana H. Nuclear field shift effect of chromium(III) in repeated extraction using a crown ether. Solvent Extr. Ion Exch. 26, 100–112 (2008).

[b26] Shibahara Y., Nishizawa K., Yasaka Y. & Fujii T. Strontium isotope effect in DMSO-water system by liquid chromatography using a cryptand polymer. Solvent Extr. Ion Exch. 20, 67–79 (2002).

[b27] Shibahara Y., Takaishi H., Nishizawa K. & Fujii T. Strontium isotope effects in ligand exchange reaction. J. Nucl. Sci. Technol. 39, 451–456 (2002).

[b28] Moynier F., Fujii T. & Albarede F. Nuclear field shift effect as a possible cause of Te isotopic anomalies in the early solar system-an alternative explanation of Fehr *et al*. (2006 and 2009). Meteorit. Planet. Sci. 44, 1735–1742 (2009).

[b29] Fujii T., Moynier F., Telouk P. & Albarede F. Nuclear field shift effect in the isotope exchange reaction of cadmium using a crown ether. Chem. Geol. 267, 157–163 (2009).

[b30] Fujii T. . Nuclear field shift effect in isotope fractionation of thallium. J. Radioanal. Nucl. Chem. 296, 261–265 (2013).

[b31] Fujii T., Moynier F., Agranier A., Ponzevera E. & Abe M. Nuclear field shift effect of lead in ligand exchange reaction using a crown ether. Proc. Radiochim. Acta 1, 387–392 (2011).

[b32] Schauble E. A. Modeling nuclear volume isotope effects in crystals. Proc. Natl. Acad. Sci. USA 110, 17714–17719 (2013). 23650350 10.1073/pnas.1216216110 PMC3816415

[b33] Zheng W. & Hintelmann H. Nuclear field shift effect in isotope fractionation of mercury during abiotic reduction in the absence of light. J. Phys. Chem. A 114, 4238–4245 (2010). 20192261 10.1021/jp910353y

[b34] Ghosh S., Schauble E. A., Couloume G. L., Blum J. D. & Bergquist B. A. Estimation of nuclear volume dependent fractionation of mercury isotopes in equilibrium liquid-vapor evaporation experiments. Chem. Geol. 336, 5–12 (2013).

[b35] Wiederhold J. G. . Equilibrium mercury isotope fractionation between dissolved Hg(II) Species and thiol-bound Hg. Environ. Sci. Technol. 44, 4191–4197 (2010). 20443581 10.1021/es100205t

[b36] Nemoto K., Abe M., Seino J. & Hada M. An ab intio study of nuclear volume effects for isotope fractionations using two-component relativistic methods. J. Comput. Chem. 36, 816–820 (2015). 25727195 10.1002/jcc.23858

[b37] Dirac P. A. M. The quantum theory of the electron. Proc. R. Soc. Series A 117, 610–624 (1928).

[b38] Dirac P. A. M. A theory of electrons and protons. Proc. R. Soc. Series A 126, 360–365 (1930).

[b39] Gratz L. E., Keeler G. J., Blum J. D. & Sherman L. S. Isotopic composition and fractionation of mercury in Great Lakes precipitation and ambient air. Environ. Sci. Technol. 44, 7764–7770 (2010). 20853890 10.1021/es100383w

[b40] Chen J. B., Hintelmann H., Feng X. B. & Dimock B. Unusual fractionation of both odd and even mercury isotopes in precipitation from Peterborough, ON, Canada. Geochim. Cosmochim. Acta 90, 33–46 (2012).

[b41] Fricke G. & Heilig K. Group I: element particles, nuclei and atoms. Nuclear charge radii, Vol. **20**, 80-Hg mercury, 1-9 (Landolt-Brnstein: numerical data and functional relationships in Science and Technology, new series, 2004).

[b42] Angeli I. A consistent set of nuclear rms charge radii: properties of the radius surface R (N, Z). Atom. Data Nucl. Data Tables 87, 185–206 (2004).

[b43] Blum J. D. & Bergquist. B. A. Reporting of variations in the natural isotopic composition of mercury. Anal. Bioanal. Chem. 388, 353–359 (2007). 17375289 10.1007/s00216-007-1236-9

[b44] Ghosh S., Xu Y. F., Humayun M. & Odom L. Mass-independent fractionation of mercury isotopes in the environment. Geochem. Geophys. Geosyst. 9, 1–10 (2008).

[b45] Estrade N., Carignan J., Sonke J. E. & Donard O. F. X. Mercury isotope fractionation during liquid-vapor evaporation experiments. Geochim. Cosmochim. Acta 73, 2693–2711 (2009).

[b46] Smith R. S. . Small-scale studies of roasted ore waste reveal extreme ranges of stable mercury isotope signatures. Geochim. Cosmochim. Acta 137, 1–17 (2014).

[b47] Basu A., Sanford R. A., Johnson T. M., Lundstrom C. C. & Löffler F. E. Uranium isotopic fractionation factors during U(VI) reduction by bacterial isolates. Geochim. Cosmochim. Acta 136, 100–113 (2014).

[b48] Bopp C. J. IV . Uranium ^238^U/^235^U isotope ratios as indicators of reduction: results from an *in situ* biostimulation experiment at Rifle, Colorado, USA. Environ. Sci. Technol. 44, 5927–5933 (2010). 20597538 10.1021/es100643v

[b49] Kleinman L. I. & Wolfsber M. Corrections to Born-Oppenheimer approximation and electronic effects on isotopic-exchange equilibria. J. Chem. Phys. 59, 2043–2053 (1973).

[b50] Kleinman L. I. & Wolfsber M. Corrections to Born-Oppenheimer approximation and electronic effects on isotopic-exchange equilibria. 2. J. Chem. Phys. 60, 4740–4748 (1974).

[b51] Miller M. F. Isotopic fractionation and the quantification of O-17 anomalies in the oxygen three-isotope system: an appraisal and geochemical significance. Geochim. Cosmochim. Acta 66, 1881–1889 (2002).

[b52] Young E. D., Galy A. & Nagahara H. Kinetic and equilibrium mass-dependent isotope fractionation laws in nature and their geochemical and cosmochemical significance. Geochim. Cosmochim. Acta 66, 1095–1104 (2002).

[b53] Cao X. B. & Liu Y. Equilibrium mass-dependent fractionation relationships for triple oxygen isotopes. Geochim. Cosmochim. Acta 75, 7435–7445 (2011).

[b54] Saue T. . DIRAC, a program for atomic and molecular direct iterative relativistic all-electron calculations, release DIRAC13.1 (2013) Available at: http://dirac.chem.sdu.dk. (Acessed: 5th January 2014).

[b55] Dyall K. G. Relativistic quadruple-zeta and revised triple-zeta and double-zeta basis sets for the 4p, 5p, and 6p elements. Theor. Chem. Acc. 115, 441–447 (2006).

[b56] Dyall K. G. & Gomes A. S. P. Revised relativistic basis sets for the 5d elements Hf-Hg. Theor. Chem. Acc. 125, 97–100 (2010).

[b57] Dunning T. H. Gaussian-basis sets for use in correlated molecular calculations. 1. The atoms boron through neon and hydrogen. J. Chem. Phys. 90, 1007–1023 (1989).

[b58] Frisch M. J. . Gaussian software package, Inc., Pittsburgh PA. Gaussian 03, Revision B.04 (2003).

[b59] Visscher L. & Dyall K. G. Dirac–Fock atomic electronic structure calculations using different nuclear charge distributions. Atom. Data Nucl. Data Tables 67, 207–224 (1997).

[b60] Richet P., Bottinga Y. & Javoy M. Review of hydrogen, carbon, nitrogen, oxygen, sulfur, and chlorine stable isotope fractionation among gaseous molecules. Ann. Rev. Earth Planet. Sci. 5, 65–110 (1977).

