# Supplementary material for: Nuclear volume effects in equilibrium stable isotope fractionations of mercury, thallium and lead
Source: Sci Rep. 2015 Jul 30;5:12626. doi: 10.1038/srep12626 (PMC4519782; doi:10.1038/srep12626)
Supplement: Supplementary Information [file srep12626-s1.doc]

**Supporting Information**

**Nuclear volume effects in equilibrium stable isotope fractionations of mercury, thallium and lead**

Sha Yang ^1, 2^ and Yun Liu ^1^*

*^1^ State Key Laboratory of Ore Deposit Geochemistry, Institute of Geochemistry, Chinese Academy of Sciences, Guiyang 550002, China*

*^2^ University of Chinese Academy of Sciences, Beijing 100049, China*

*: Corresponding author (Email: Liuyun@vip.gyig.ac.cn)

**Table S1**. Optimized structures of Hg, Tl, and Pb molecules*

| Molecule | Symmetry | Hg-X bond length (Å) | |
| --- | --- | --- | --- |
|  |  | HF (Gaussian 03) | DHF (DIRAC 13.1) |
| HgCl_2_ | D_∞h_ | 2.3002 | 2.2912 |
| HgBr_2_ | D_∞h_ | 2.4346 | 2.4224 |
| Hg(CH_3_)Cl | C_3v_ | 2.1040(C), 2.3417(Cl) | 2.0949(C), 2.3284(Cl) |
| Hg(CH_3_)_2_ | D_3h_ | 2.1280 | 2.1181 |
| HgCl_3_^-^ | D_3h_ | 2.4700 | 2.4584 |
| HgCl_4_^2-^ | T_d_ | 2.6143 | 2.6018 |
| HgBr_3_^-^ | D_3h_ | 2.6005 | 2.5856 |
| HgBr_4_^2-^ | T_d_ | 2.7507 | 2.7337 |
| Hg(H_2_O)_6_^2+^ | T_h_ | 2.4091 | 2.4025 |
| Hg(OH)_2_ | C_2_, C_2h_ | 1.9643 | 1.9571 |
|  |  | Tl-X bond length (Å) | |
|  |  | HF (Gaussian 03) | DHF (DIRAC 13.1) |
| Tl(H_2_O)^3+^ | C_3_ | 2.7677 | 2.7522 |
| Tl(H2O)_6_^3+^ | T_h_ | 2.2730 | 2.2560 |
|  |  | Pb-X bond length (Å) | |
|  |  | HF (Gaussian 03) | DHF (DIRAC 13.1) |
| PbCl_4_^2-^ | T_d_ | 2.8910 | 2.8757 |
| PbBr_4_^2-^ | T_d_ | 3.0449 | 3.0258 |
| PbCl_4_ | T_d_ | 3.3921 | 3.3639 |

*: DHF bond lengths are generally found to be about 0.01-0.02Å shorter than HF bond lengths. For Hg(OH)_2_ species, we use C_2_ symmetry for calculating harmonic vibrational frequencies by Gaussian 03 and C_2h_ symmetry for calculating total energies by Dirac13.1.

**Table S2.** Vibrational frequencies (*v*) for the calculation of conventional mass-dependent fractionation factors of Hg-, Tl- and Pb-bearing species

| Calculated harmonic vibrational frequencies (cm^-1^) | | | | | |
| --- | --- | --- | --- | --- | --- |
| Molecule | *v*(^202^HgX) | *v*(^198^HgX) | Molecule | *v*(^202^HgX) | *v*(^198^HgX) |
| HgCl_2_ | 93.4373^*^ | 93.6779^*^ | HgBr_2_ | 64.2650^*^ | 64.5471^*^ |
|  | 346.8154 | 346.8154 |  | 215.9938 | 215.9938 |
|  | 400.9009 | 401.9335 |  | 285.0655 | 286.3166 |
| Hg(CH_3_)Cl | 122.0160^*^ | 122.2169^*^ | Hg(H_2_O)_6_^2+^ | 40.2282^**^ | 40.2302^**^ |
|  | 343.6647 | 344.0642 |  | 57.0408^**^ | 57.0408^**^ |
|  | 548.6489 | 549.1008 |  | 60.4317^**^ | 60.5621^**^ |
|  | 838.9791^*^ | 839.0109^*^ |  | 152.3932^*^ | 152.3932^*^ |
|  | 1327.3203 | 1327.3204 |  | 194.8228^*^ | 194.8228^*^ |
|  | 1559.4827^*^ | 1559.4833^*^ |  | 203.6238^**^ | 203.6238^**^ |
|  | 3194.7021 | 3194.7021 |  | 252.2596^**^ | 252.5559^**^ |
|  | 3296.4554^*^ | 3296.4554^*^ |  | 280.3187 | 280.3187 |
| Hg(CH_3_)_2_ | 22.8722 | 22.8722 |  | 302.9292 | 302.9292 |
|  | 150.3489^*^ | 150.5308^*^ |  | 354.6421^**^ | 354.6421^**^ |
|  | 517.0593 | 517.0593 |  | 378.3490^**^ | 378.3613^**^ |
|  | 545.1915 | 545.8986 |  | 506.1910^**^ | 506.2271^**^ |
|  | 728.9048^*^ | 728.9048^*^ |  | 508.1310^**^ | 508.1310^**^ |
|  | 822.2479^*^ | 822.3193^*^ |  | 1776.1281^*^ | 1776.1281^*^ |
|  | 1323.2445 | 1323.2453 |  | 1777.7488^**^ | 1777.7491^**^ |
|  | 1324.4974 | 1324.4974 |  | 1783.2612 | 1783.2612 |
|  | 1558.1867^*^ | 1558.1867^*^ |  | 4043.9835^*^ | 4043.9835^*^ |
|  | 1561.5765^*^ | 1561.5775^*^ |  | 4045.4644^**^ | 4045.4644^**^ |
|  | 3161.7663 | 3161.7664 |  | 4053.8064 | 4053.8064 |
|  | 3164.2507 | 3164.2507 |  | 4132.8763^**^ | 4132.8763^**^ |
|  | 3246.2840^*^ | 3246.2840^*^ |  | 4133.4243^**^ | 4133.4243^**^ |
|  | 3246.6380^*^ | 3246.6380^*^ | HgBr_3_^-^ | 48.0775^*^ | 48.1756^*^ |
| HgCl_3_^-^ | 66.5221^*^ | 66.6318^*^ |  | 61.9376 | 62.2718 |
|  | 82.5285 | 82.8109 |  | 174.1759 | 174.1759 |
|  | 264.3558^*^ | 264.8240^*^ |  | 183.4239^*^ | 184.0380^*^ |
|  | 279.2627 | 279.2627 | Hg(OH)_2_ | 137.8670 | 137.8805 |
| HgCl_4_^2-^ | 64.3302^*^ | 64.3302^*^ |  | 177.8575 | 178.1062 |
|  | 85.7310^**^ | 85.9507^**^ |  | 179.0747 | 179.3116 |
|  | 185.5904^**^ | 185.8738^**^ |  | 607.9784 | 607.9788 |
|  | 226.5430 | 226.5430 |  | 673.8993 | 674.8711 |
| HgBr_4_^2-^ | 41.5881^*^ | 41.5881^*^ |  | 937.8138 | 937.8357 |
|  | 60.8467^**^ | 61.0245^**^ |  | 949.8887 | 949.9102 |
|  | 125.0627^**^ | 125.4584^**^ |  | 4142.7930 | 4142.7931 |
|  | 140.8241 | 140.8241 |  | 4143.5436 | 4143.5436 |
| Molecule | *v*(^205^TlX) | *v*(^203^TlX) | Molecule | *v*(^205^TlX) | *v*(^203^TlX) |
| Tl(H_2_O)^3+^ | 59.0256^*^ | 59.0329^*^ | Tl(H_2_O)_6_^3+^ | 73.2808^**^ | 73.2813^**^ |
|  | 70.0299 | 70.0682 |  | 100.9427^**^ | 101.0512^**^ |
|  | 148.1320^*^ | 148.1562^*^ |  | 107.7514^**^ | 107.7514^**^ |
|  | 177.9058 | 177.9854 |  | 186.4456^*^ | 186.4456^*^ |
|  | 245.0916^*^ | 245.0940^*^ |  | 250.6516^**^ | 250.6516^**^ |
|  | 327.4715 | 327.4720 |  | 301.8368^*^ | 301.8368^*^ |
|  | 369.1792* | 369.1805^*^ |  | 350.6765 | 350.6765 |
|  | 388.2646 | 388.2766 |  | 364.7318^**^ | 364.9381^**^ |
|  | 419.6240^*^ | 419.6273^*^ |  | 411.2460 | 411.2460 |
|  | 504.9587 | 504.9590 |  | 489.4982^**^ | 489.4982^**^ |
|  | 1759.9540 | 1759.9540 |  | 509.3056^**^ | 509.3161^**^ |
|  | 1761.3247^*^ | 1761.3247^*^ |  | 663.3222^**^ | 663.3222^**^ |
|  | 4069.8011 | 4069.8011 |  | 668.3897^**^ | 668.4113^**^ |
|  | 4072.1939^*^ | 4072.1939^*^ |  | 1778.0195^*^ | 1778.0195^*^ |
|  | 4167.8464 | 4167.8464 |  | 1782.9159^**^ | 1782.9161^**^ |
|  | 4168.3872^*^ | 4168.3872^*^ |  | 1792.8409 | 1792.8409 |
|  |  |  |  | 3916.8684^*^ | 3916.8684^*^ |
|  |  |  |  | 3921.8088^**^ | 3921.8088^**^ |
|  |  |  |  | 3942.8503 | 3942.8503 |
|  |  |  |  | 3997.9329^**^ | 3997.9329^**^ |
|  |  |  |  | 3999.2664^**^ | 3999.2664^**^ |
| Molecule | *v*(^208^PbX) | *v*(^207^PbX) | *v*(^206^PbX) |  |  |
| PbCl_4_^2-^ | 29.2439^**^ | 29.2511^**^ | 29.2583^**^ |  |  |
|  | 54.4843^*^ | 54.4843^*^ | 54.4843^*^ |  |  |
|  | 152.2528^**^ | 152.3632^**^ | 152.4747^**^ |  |  |
|  | 195.7801 | 195.7801 | 195.7801 |  |  |
| PbBr_4_^2-^ | 22.1504^**^ | 22.1563^**^ | 22.1621^**^ |  |  |
|  | 34.1430^*^ | 34.1430^*^ | 34.1430^*^ |  |  |
|  | 111.2159^**^ | 111.3485^**^ | 111.4823^**^ |  |  |
|  | 122.3040 | 122.3040 | 122.3040 |  |  |
| PbCl_4_ | 89.7973^*^ | 89.7973^*^ | 89.7973^*^ |  |  |
|  | 110.2515^**^ | 110.3125^**^ | 110.3739^**^ |  |  |
|  | 354.1633 | 354.1633 | 354.1633 |  |  |
|  | 372.5006^**^ | 372.6563^**^ | 372.8137^**^ |  |  |

* are doubly degenerate, ** are triply degenerate.

**Table S3**. Calculated total energies (i.e., electron energies plus nuclear repulsion energies) of Hg-bearing species

| Species | E(^202^Hg) (Hartree) | E(^198^Hg) (Hartree) |
| --- | --- | --- |
| Hg^0^ | -19648.872900452 | -19648.899279761 |
| Hg^2+^ | -19647.928339749 | -19647.954715647 |
| HgCl_2_ | -20570.839876388 | -20570.866254331 |
| HgBr_2_ | -24858.786154305 | -24858.812532295 |
| Hg(CH_3_)Cl | -20149.473020068 | -20149.499398518 |
| Hg(CH_3_)_2_ | -19728.085945293 | -19728.112323991 |
| HgCl_3_^-^ | -21031.911516436 | -21031.937893728 |
| HgCl_4_^2-^ | -21492.847297638 | -21492.873674561 |
| HgBr_3_^-^ | -27463.831015157 | -27463.857392566 |
| HgBr_4_^2-^ | -30068.742826148 | -30068.769203205 |
| Hg(H_2_O)_6_^2+^ | -20104.905854632 | -20104.932230959 |
| Hg(OH)_2_ | -19799.820912939 | -19799.847291190 |

**Table S4**. Calculated total energies (i.e., electron energies plus nuclear repulsion energies) of Tl-bearing species

| Species | E(^205^Tl) (Hartree) | E(^203^Tl) (Hartree) |
| --- | --- | --- |
| Tl^0^ | -20274.868664124 | -20274.883796548 |
| Tl^+^ | -20274.686555259 | -20274.701687902 |
| Tl^3+^ | -20272.974422708 | -20272.989552730 |
| Tl(H_2_O)_3_^+^ | -20503.029404493 | -20503.044537094 |
| Tl(H_2_O)_6_^3+^ | -20730.389778832 | -20730.404909414 |

**Table S5**. Calculated total energies (i.e., electron energies plus nuclear repulsion energies) of Pb-bearing species

| Species | E(^208^Pb) (Hartree) | E(^207^Pb) (Hartree) | E(^206^Pb) (Hartree) |
| --- | --- | --- | --- |
| Pb^0^ | -20913.734134618 | -20913.745577969 | -20913.753146160 |
| Pb^2+^ | -20913.022449627 | -20913.033893321 | -20913.041461739 |
| Pb^4+^ | -20910.434076761 | -20910.445517998 | -20910.453084792 |
| PbCl_4_^2-^ | -22757.807024151 | -22757.818467855 | -22757.826036280 |
| PbBr_4_^2-^ | -31333.699170932 | -31333.710614620 | -31333.718183034 |
| PbCl_4_ | -22757.700613181 | -22757.712055724 | -22757.719623382 |

**Table S6**. Comparison of the results using the same theoretical method and nuclear charge radii

|  | DIRAC13.1 (This study) | | | | | DIRAC04^a)^ | | | | |
| --- | --- | --- | --- | --- | --- | --- | --- | --- | --- | --- |
|  | 0℃ | 25℃ | 100℃ | 300℃ | 1000℃ | 0℃ | 25℃ | 100℃ | 300℃ | 1000℃ |
| Hg^0^ | 0 | 0 | 0 | 0 | 0 | 0 | 0 | 0 | 0 | 0 |
| Hg^2+^ | 3.47 | 3.18 | 2.54 | 1.65 | 0.74 | 3.47 | 3.17±0.6 | 2.54 | 1.65 | 0.74 |
| HgCl_2_ | 1.39 | 1.27 | 1.02 | 0.66 | 0.30 | 1.39 | 1.27±0.3 | 1.02 | 0.66 | 0.30 |
| HgBr_2_ | 1.34 | 1.23 | 0.98 | 0.64 | 0.29 | 1.34 | 1.23±0.2 | 0.98 | 0.64 | 0.29 |
| Hg(CH_3_)Cl | 0.87 | 0.80 | 0.64 | 0.42 | 0.19 | 0.88 | 0.80±0.2 | 0.64 | 0.42 | 0.19 |
| Hg(CH_3_)_2_ | 0.62 | 0.57 | 0.46 | 0.30 | 0.13 | 0.62 | 0.57±0.1 | 0.45 | 0.30 | 0.13 |
| HgCl_4_^2-^ | 2.43 | 2.22 | 1.78 | 1.16 | 0.52 | 2.42 | 2.22±0.4 | 1.77 | 1.16 | 0.52 |
| Hg(H_2_O)_6_^2+^ | 3.03 | 2.78 | 2.22 | 1.44 | 0.65 | 3.01 | 2.75±0.6 | 2.20 | 1.43 | 0.64 |

a) Calculated with the software package DIRAC04 by Schauble^1^.

Table S6 shows the comparison results using same theoretical method (i.e., Dirac-Hartree-Fock (DHF) theory) and nuclear charge radii (i.e., from Angeli^2^) between Schauble^1^ and this study. It also shows different versions of the software package (i.e., DIRAC04 and DIRAC13.1) have little impact on the calculation results.

Reference

1.Schauble, E. A. Role of nuclear volume in driving equilibrium stable isotope fractionation of mercury, thallium, and other very heavy elements. *Geochim. Cosmochim. Acta* **71**, 2170-2189 (2007).

2. Angeli, I. A consistent set of nuclear rms charge radii: properties of the radius surface R (N, Z). *Atom. Data Nucl. Data Tables* **87**, 185-206 (2004).
